# Synthesis and crystal structures of two purpurin derivatives: 1,4-dihy­droxy-2-propoxyanthra­quinone and 2-but­oxy-1,4-di­hydroxy­anthra­quinone

**DOI:** 10.1107/S2056989017014724

**Published:** 2017-10-20

**Authors:** Eric Bosch, Emily N. McClain

**Affiliations:** a Department of Chemistry, Missouri State University, Springfield, MO 65897, USA

**Keywords:** crystal structure, purpurin, anthra­quinone, hydrogen bonding

## Abstract

The title compounds were obtained by deprotonation of 1,2,4-tri­hydroxy­anthra­quinone (purpurin) using sodium hydride followed by reaction with either 1-bromo­propane or 1-bromo­butane. 1,4-Dihy­droxy-2-propoxyanthra­quinone crystallizes as a 1:1 solvate from aceto­nitrile, C_17_H_14_O_5_·CH_3_CN. The anthra­quinone core of the mol­ecule is essentially planar and both hy­droxy groups participate in intra­molecular O—H⋯O (carbon­yl) hydrogen bonds. The propyl chain is angled slightly above the plane of the anthra­quinone moiety with a maximum deviation of 0.247 (2) Å above the plane for the terminal carbon atom. In contrast, 2-but­oxy-1,4-di­hydroxy­anthra­quinone, C_18_H_16_O_5_, crystallizes from nitro­methane with two independent mol­ecules in the asymmetric unit. The anthra­quinone core of each independent mol­ecule is essentially planar and both hy­droxy groups on both mol­ecules participate in intra­molecular O—H⋯O(carbon­yl) hydrogen bonds. The butyl chain in one mol­ecule is also angled slightly above the plane of the anthra­quinone moiety, with a maximum deviation of 0.833 (5) Å above the plane for the terminal carbon atom. In contrast, the butyl group on the second mol­ecule is twisted out of the plane of the anthra­quinone core with a torsion angle of 65.1 (3)°, resulting in a maximum deviation of 1.631 (5) Å above the plane for the terminal carbon atom.

## Abstract 2

The title compounds were obtained by deprotonation of 1,2,4-tri­hydroxy­anthra­quinone (purpurin) using sodium hydride followed by reaction with either 1-bromo­propane or 1-bromo­butane. The compounds were characterized by ^1^H and ^13^C NMR and the structures determined using single-crystal X-ray diffraction.

## Chemical context   

Purpurin, 1,2,4-trihy­droxy anthra­quinone, is a major component of the dye extracted from madder root (Schweppe & Winter, 1997 ▸). The extract from madder root has been used to dye wool and other fabrics since anti­quity. Purpurin is commercially available and we here report two derivatives, 1,4-dihy­droxy-2-prop­oxy anthra­quinone and 2-but­oxy-1,4-dihy­droxy anthra­quinone, prepared by selective deprotonation of purpurin followed by alkyl­ation with the either 1-bromo­propane or 1-bromo­butane.
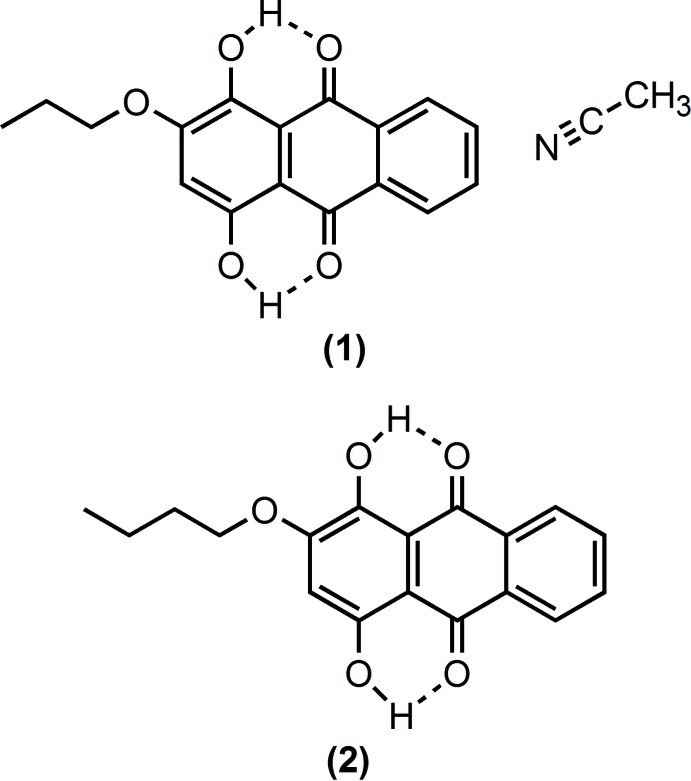


## Structural commentary   

**Figure 1:**
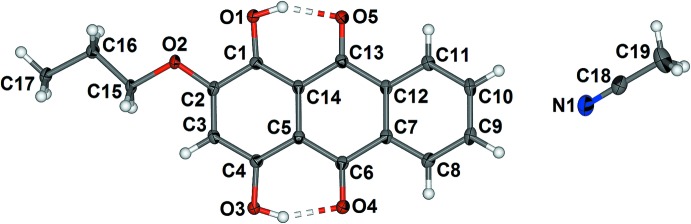
Mol­ecular structure of (1) with the included aceto­nitrile. Displacement ellipsoids of non-H atoms are drawn at the 50% probability level and H atoms are shown as circles of arbitrary size.

The asymmetric unit of 1,4-dihy­droxy-2-prop­oxy anthra­quinone (1), crystallized from aceto­nitrile solvent, contains a single anthra­quinone mol­ecule and one aceto­nitrile solvate mol­ecule as shown in Fig. 1 ▸. The two intra­molecular hydrogen bonds (Table 1 ▸) are typical for the 1,4-dihy­droxy anthra­quinones and 1-hy­droxy­anthra­quinones. These hydrogen bonds are maintained in chloro­form solution, as shown by the chemical shift of 13.47 and 13.56 ppm for the two hydroxyl protons. The anthra­quinone moiety is planar, with an average root mean square (r.m.s.) deviation of atoms C1 to C14 of 0.021 Å, in which the maximum deviation from the plane defined by atoms C1 to C14 is 0.044 (1) Å for C9. The propyl chain is angled slightly above the plane of the anthra­quinone moiety, with deviations of 0.043 (2), 0.143 (2) and 0.247 (2) Å for atoms C15, C16 and C17, respectively, from the plane defined by atoms C1–C14. The aceto­nitrile is angled towards H10 with a N1⋯C10 distance of 3.401 (2) Å. The final difference map shows several peaks of 0.2 to 0.7 e Å^−3^ in the anthra­quinone plane that suggest the presence of minor whole-mol­ecule disorder in which the anthra­quinone is translated in the plane and/or flipped over.

**Figure 2:**
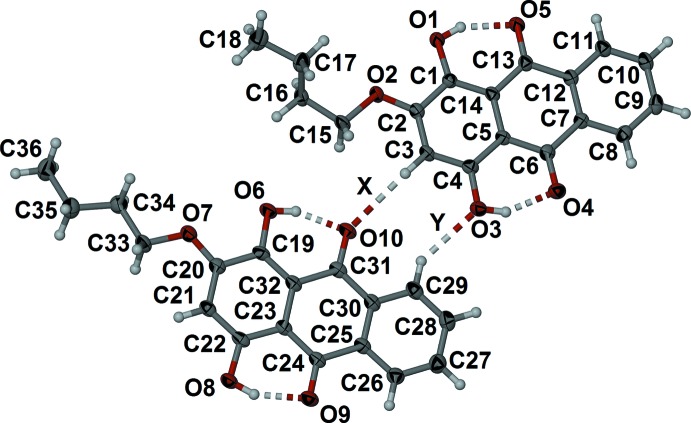
Asymmetric unit of (2) showing the close inter­molecular C—H⋯O contacts X and Y (see text). Displacement ellipsoids of non-H atoms are drawn at the 50% probability level and H atoms are shown as circles of arbitrary size.

In contrast, the asymmetric unit of 2-but­oxy-1,4-dihy­droxy anthra­quinone (2) crystallized from nitro­methane solvent, contains two unique anthra­quinone mol­ecules as shown in Fig. 2 ▸. Both mol­ecules feature two intra­molecular hydrogen bonds (Table 2 ▸) similar to those observed in (1). These hydrogen bonds are also maintained in chloro­form solution, as shown by the chemical shift of 13.46 and 13.55 ppm for the two hydroxyl protons. The anthra­quinone moieties in both mol­ecules are planar. The r.m.s deviation of atoms C1 to C14 is 0.006 Å, with a maximum deviation from the plane defined by atoms C1 to C14 of 0.011 (2) Å for C13. The r.m.s. deviation from the plane defined by atoms C19 to C32 is 0.025 Å, with a maximum deviation of 0.048 (2) Å for C31. The butyl chain attached to O2 is twisted out of the C1–C14 anthra­quinone plane with a O2—C15—C16—C17 torsion angle of −65.1 (3)°. The butyl chain has an *anti*-conformation, the C15—C16—C17—C18 torsion angle being −173.1 (2)°. The deviations of the butyl carbon atoms from the anthra­quinone plane defined by atoms C1 to C14 are 0.101 (4), 0.194 (4), 1.467 (4) and 1.631 (5) Å for atoms C15, C16, C17 and C18, respectively. The butyl chain in the second unique mol­ecule, attached to O7, is tilted slightly out of the plane of the anthra­quinone with a C20—O7—C33—C34 torsion angle of −167.3 (2)°. This butyl chain also adopts an *anti*-conformation, the C33—C34—C35—C36 torsion angle being −175.2 (3)°. The resultant deviations of the butyl carbon atoms from the plane defined by atoms C19–C32 are 0.077 (4), 0.428 (4), 0.356 (4) and 0.833 (5) Å for atoms C33, C34, C35 and C36, respectively. There is a close inter­molecular contact between phenyl hydrogen atom H3 and carbonyl oxygen atom O10, with a C3⋯O10 distance of 3.494 (3) Å (labelled X in Fig. 2 ▸). A second close inter­molecular contact, between phenyl hydrogen atom H29 and hydroxyl oxygen atom O3 gives a C29⋯O3 distance of 3.231 (4) Å (labelled Y in Fig. 2 ▸).

## Supra­molecular features   

**Figure 3:**
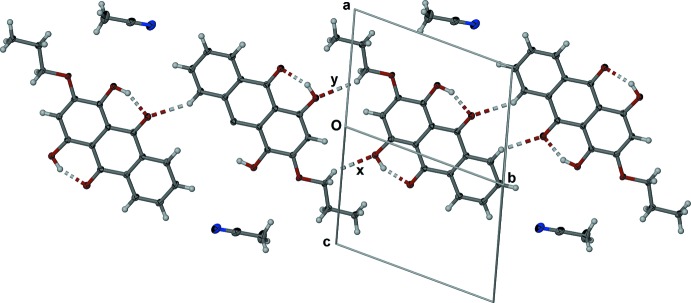
Structure of (1) viewed along the [101] direction, with close C—H⋯O contacts labelled x and y (see text).

**Figure 4:**
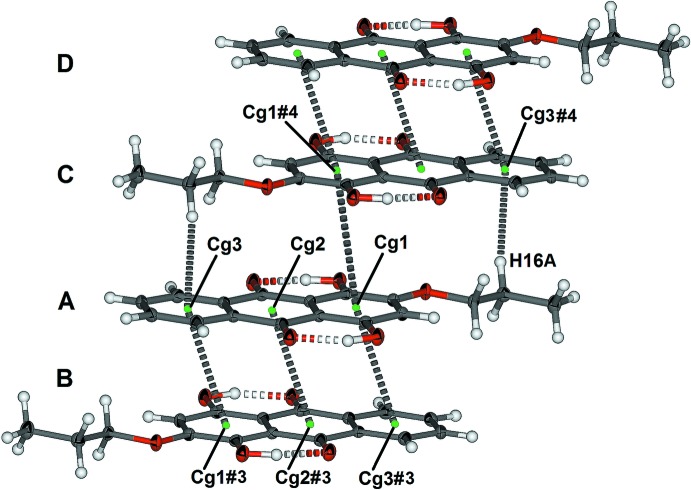
Repetitive π-stacking of (1). Displacement ellipsoids of non-H atoms are drawn at the 50% probability level [symmetry codes: (#1) − *x*, −*y*, 1 − *z*; (#2) 2 − *x*, 2 − *y*, 2 − *z*; (#3) 1 − *x*, 1 − *y*, 1 − *z*; (#4) 2 − *x*, 1 − *y*, 1 − *z*].

In the crystal, mol­ecules of (1) form planes that incorporate the aceto­nitrile mol­ecule, as shown in Fig. 3 ▸. The aceto­nitrile mol­ecule is almost coplanar with the anthra­quinone moiety, with deviations of 0.401 (2), 0.536 (2) and 0.722 (2) Å for atoms N1, C18, and C19, respectively, from the plane defined by atoms C1–C14. There is a close C—H⋯O inter­action (Table 1 ▸) between a phenyl hydrogen atom and an adjacent carbonyl oxygen atom of an inversion-related mol­ecule of (1). The C11⋯O5#2 distance is 3.245 (2) Å [symmetry code: (#2) 2 − *x*, 2 − *y*, 1 − *z*] and the inter­action is labelled x in Fig. 3 ▸. The methyl­ene hydrogen H15*A* is close to the carbonyl oxygen O3 of a second inversion-related mol­ecule of (1). The C15⋯O3#1 distance is 3.218 (2) Å [symmetry code: (#1) 1 − *x*, −*y*, 1 − *z*], and the inter­action is labelled y in Fig. 3 ▸. The anthra­quinone units of (1) alternately π-stack in pairs as shown in Fig. 4 ▸. Each π-stacked pair (A and B in Fig. 4 ▸) has significant overlap of the anthra­quinone moiety with *Cg*1⋯*Cg*3#3, *Cg*2⋯*Cg*2#3 [symmetry code: (#3) 1 − *x*, 1 − *y*, 1 − *z*; *Cg*1, *Cg*2 and *Cg*3 are the centroids of the six-membered rings C1–C5/C14, C5–C7/C12–C14 and C7–C12, respectively] distances of 3.607 (1) and 3.569 (1) Å, respectively, with slippages of 1.304 and 1.331 Å, respectively. The pairs of π-stacked mol­ecules of (1) are offset π-stacked and the alkyl chain has a C—H⋯π inter­action with one end of the anthra­quinone unit, as shown in Fig. 4 ▸ (mol­ecules labelled A and C). The C16⋯*Cg*3#4 distance is 3.587 (2) Å [symmetry code: (#4) 2 − *x*, 1 − *y*, 1 − *z*].

**Figure 5:**
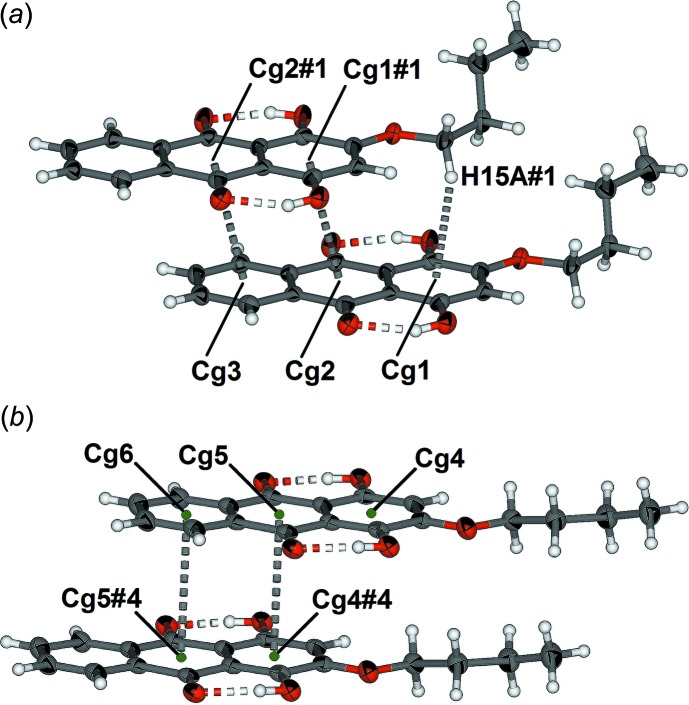
π-Stacking of the two unique mol­ecules of (2) showing the C—H⋯π and π–π inter­actions as grey dashed lines. Part (*a*) shows the C1–C14 anthra­quinone unit and (*b*) the C19–C32 anthra­quinone unit. Displacement ellipsoids drawn at the 50% probability level [symmetry codes: (#1) *x* − 1, *y z*; (#2) 3 − *x*, 1 − *y*, 1 − *z*; (#3) 2 − *x*, 1 − *y*, 1 − *z*; (#4) 1 + *x*, *y*, *z*].

The two unique anthra­quinone mol­ecules in the asymmetric unit of (2) offset π-stack in individual columns. There are three close C—H⋯O contacts (Table 2 ▸) between these offset π-stacked columns. The C⋯O distances are 3.485 (3), 3.502 (3) and 3.548 (4) Å for C15⋯O6#1, C21⋯O8#2, and C33⋯O9#3. respectively [symmetry codes: (#1) *x* − 1, *y*, *z*; (#2) 3 − *x*, 1 − *y*, 1 − *z*; (#3) 2 − *x*, 1 − *y*, 1 − *z*]. The inter­actions within each of the two unique sets of π-stacked mol­ecules are shown in Fig. 5 ▸. For the anthra­quinone unit defined by C1–C14 (Fig. 5 ▸ *a*), the centroid-to-centroid distances *Cg*2⋯*Cg*1#1 and *Cg*3⋯*Cg*2#1 are 3.521 (2) and 3.517 (2) Å, with slippages of 0.960 and 0.948 Å, respectively, where *Cg*1, *Cg*2 and *Cg*3 are the centroids of the six-membered rings C1–C5/C14, C5–C7/C12–C14 and C7–C12, respectively. The methyl­ene hydrogen atom H15*A*#1 is positioned above centroid *Cg*1 with a C15⋯*Cg*1 distance of 3.690 (3) Å. For the anthra­quinone unit defined by C19–C32 (Fig. 5 ▸ *b*), the centroid-to-centroid distances *Cg*5⋯*Cg*4#4 and *Cg*6⋯*Cg*5#4 [symmetry code: (#4) 1 + *x*, *y*, *z*; *Cg*4, *Cg*5 and *Cg*6 are the centroids of the C19–C23/C32, C23—C25/C30-C32 and C25–C30 rings, respectively] are 3.520 (1) and 4.009 (1) Å with slippages of 0.960 and 2.145 Å, respectively.

## Database survey   

A search of the Cambridge Crystallographic Database (Version 5.38, Nov. 2016; Groom *et al.*, 2016 ▸) using *Conquest* (Bruno *et al.*, 2002 ▸) for the anthra­quinone ring system with oxygen atoms at positions 1, 2 and 4 without restriction on substitution of the other aromatic position, revealed 15 structures. Database entries not including atomic coordinates were excluded. The structure of the parent compound, 1,2,4-trihy­droxy anthra­quinone monohydrate has been reported (refcode QEGNEV; Yatsenko *et al.*, 2000 ▸). In addition, structures have been determined for several organic derivatives that were isolated from natural sources. For example, the derivative most closely related to the structures reported here, 1,4-dihy­droxy-2-meth­oxy-7-methyl­anthracene-9,10-dione, has been isolated from two different fungi and the structure reported [refcodes GEPCOU (She *et al.*, 2006 ▸) and GEPCOU01 (Muangsin *et al.*, 2008 ▸)]. Complexes of purpurin with rhenium (refcodes CEVNIB, CEVNOH and AVABEF; Sathiyendiran, *et al.*, 2006 ▸, 2011 ▸), copper [refcode ZOMSEB; Das, *et al.*, 2014 ▸), tin (refcodes MOQTAO and MOQTES; de Sousa *et al.*, 2009 ▸), calcium and aluminum (refcode LAYBAO; Bergerhoff & Wunderlich, 1993 ▸) have been reported. In each of the reported structures, those compounds with a free hydroxyl group flanking the anthra­quinone carbonyl also exhibit the intra­molecular hydrogen bond reported for (1) and (2).

## Synthesis and crystallization   

*Synthesis of 1,4-dihy­droxy-2-prop­oxy anthra­quinone (1).* In a flask under an atmosphere of argon, a dark red–orange solution of purpurin (0.26 g) in di­methyl­formamide (10 mL) and tetra­hydro­furan (20 mL) was cooled in an ice–salt bath. Sodium hydride (0.081 g, 1 eq.) was added and the resultant violet solution was stirred in the ice bath for 20 minutes. Excess 1-bromo­propane (1 mL) was added, a water condenser attached, and the flask was removed from the cooling bath and heated to 353 K for 24 h. The flask was cooled to room temperature and the solvents evaporated. The crude product was purified by column chromatography with silica gel (0.65–0.40 mm) and mixtures of hexane and ethyl acetate of increasing polarity. The eluant was monitored by TLC with a 5:1 mixture of hexane and ethyl acetate. The solvent was evaporated and the product obtained as a red–orange solid (0.15 g). ^1^H NMR: (400MHz, CDCl_3_) δ 13.56 (*s*, 1H), 13.47 (*s*, 1H), 8.33 (*dd*, *J* = 2.0, 7.0 Hz, 2H), 7.84–7.77 (*m*, 2H), 6.67 (*s*, 1H), 4.09 (*t*, *J* = 8.0 Hz, 2H), 1.97 (*s*, *J* = 7.0 Hz, 2H), 1.11 (*t*, *J* = 7.4 Hz, 3H). ^13^C NMR: 189.87, 186.98, 163.64, 159.97, 153.30, 137.17, 136.78, 136.41, 135.95, 129.63, 129.47, 115.07, 110.03, 108.64, 73.84, 24.68, 13.02. Compound (1) crystallized from aceto­nitrile as large dark-red blocks that included an aceto­nitrile molecule as a 1:1 solvate. When these blocks were cut to small individual pieces or ground with a mortar and pestle they appeared orange. The crystals lost luster after removal from the mother liquor, presumably due to loss of the aceto­nitrile.

*Synthesis of 4-but­oxy-1,2-di­hydroxy­anthra­quinone (2).* The same procedure was used with 1 mL of 1-bromo­butane. The compound was isolated as a dark red–purple solid. ^1^H NMR: (400MHz, CDCl_3_) δ 13.55 (*s*, 1H), 13.46 (*s*,1H), 8.33 (*dd*, *J* = 2.0, 7.0 Hz, 2H), 7.84–7.76 (*m*, 2H), 6.66 (*s*, 1H), 4.13 (*t*, *J* = 6.6 Hz, 2H), 1.92 (*m*, 2H), 1.56 (*m*, 2H), 1.02 (*t*, *J* = 7.4 Hz, 3H). ^13^C NMR: 187.40, 184.50, 161.22, 157.56, 150.87, 134.72, 134.33, 133.96, 133.51, 107.57, 106.18, 69.73, 30.85, 19.37, 13.98. Compound (2) was recrystallized from nitro­methane as dark red–black blocks. When these blocks were cut to small individual pieces or ground with a mortar and pestle they appeared orange–red.

## Refinement   

Crystal data, data collection and structure refinement details are summarized in Table 3 ▸. Hydrogen atoms potentially involved in hydrogen-bonding inter­actions were located by difference methods and initially restrained in the refinement with O—H = 0.84 (2) Å and with *U*_iso_(H) = 1.2*U*_eq_(O). Other H atoms were included in the refinement at calculated positions, C—H = 0.95 Å for aromatic, C—H = 0.99 Å for methyl­ene and C—H = 0.98 Å for methyl hydrogens with *U*_iso_(H) = 1.2*U*_eq_(C) for aromatic and methyl­ene H atoms and 1.5*U*_eq_(C) for methyl H atoms.

## Supplementary Material

Crystal structure: contains datablock(s) 1, 2, global. DOI: 10.1107/S2056989017014724/pk2605sup1.cif

Structure factors: contains datablock(s) 1. DOI: 10.1107/S2056989017014724/pk26051sup2.hkl

Supporting information file. DOI: 10.1107/S2056989017014724/pk26051sup4.cdx

Structure factors: contains datablock(s) 2. DOI: 10.1107/S2056989017014724/pk26052sup3.hkl

Supporting information file. DOI: 10.1107/S2056989017014724/pk26051sup5.cml

CCDC references: 1579381, 1579380

Additional supporting information:  crystallographic information; 3D view; checkCIF report

## Figures and Tables

**Table 1:** Hydrogen-bond geometry (Å, °) for (1)

*D*—H⋯*A*	*D*—H	H⋯*A*	*D*⋯*A*	*D*—H⋯*A*
O1—H1*O*⋯O5	0.88 (1)	1.75 (1)	2.5537 (13)	152 (2)
O3—H3*O*⋯O4	0.87 (1)	1.75 (1)	2.5578 (13)	153 (2)
C10—H10⋯N1	0.95	2.73	3.4009 (19)	128
C15—H15*A*⋯O3^i^	0.99	2.57	3.2179 (16)	123
C11—H11⋯O5^ii^	0.95	2.47	3.2446 (17)	138

Symmetry codes: (i) 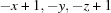; (ii) 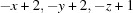.

**Table 2:** Hydrogen-bond geometry (Å, °) for (2)

*D*—H⋯*A*	*D*—H	H⋯*A*	*D*⋯*A*	*D*—H⋯*A*
O1—H1*O*⋯O5	0.85 (2)	1.78 (2)	2.564 (3)	153 (3)
O3—H3*O*⋯O4	0.87 (2)	1.74 (2)	2.536 (3)	151 (3)
O6—H6*O*⋯O10	0.86 (2)	1.72 (2)	2.542 (3)	158 (3)
O8—H8*O*⋯O9	0.87 (2)	1.73 (2)	2.554 (3)	155 (3)
C3—H3⋯O10	0.95	2.55	3.494 (3)	173
C29—H29⋯O3	0.95	2.41	3.231 (4)	144
C15—H15*B*⋯O6^i^	0.99	2.52	3.485 (3)	164
C21—H21⋯O8^ii^	0.95	2.55	3.502 (3)	180
C33—H33*A*⋯O9^iii^	0.99	2.56	3.547 (4)	172

Symmetry codes: (i) 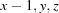; (ii) 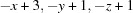; (iii) 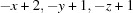.

**Table 3:** Experimental details

	(1)	(2)
Crystal data		
Chemical formula	C_17_H_14_O_5_·C_2_H_3_N	C_18_H_16_O_5_
*M*_r_	339.33	312.31
Crystal system, space group	Triclinic, *P* 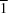	Monoclinic, *P*2_1_/*n*
Temperature (K)	100	100
*a*, *b*, *c* (Å)	8.2160 (11), 9.8605 (13), 10.7410 (14)	4.7730 (9), 44.272 (8), 13.807 (3)
α, β, γ (°)	95.999 (2), 90.181 (2), 113.774 (2)	90, 95.164 (2), 90
*V* (Å^3^)	790.99 (18)	2905.8 (9)
*Z*	2	8
Radiation type	Mo *K*α	Mo *K*α
μ (mm^−1^)	0.10	0.10
Crystal size (mm)	0.45 × 0.18 × 0.09	0.48 × 0.10 × 0.03

Data collection		
Diffractometer	Bruker APEXII CCD	Bruker APEXII CCD
Absorption correction	Multi-scan (*SADABS*; Bruker, 2014 ▸)	Multi-scan (*SADABS*; Bruker, 2014 ▸)
*T*_min_, *T*_max_	0.869, 1.000	0.854, 1.000
No. of measured, independent and observed [*I* > 2σ(*I*)] reflections	10216, 3548, 2718	36901, 6456, 3747
*R*_int_	0.022	0.104
(sin θ/λ)_max_ (Å^−1^)	0.644	0.643

Refinement		
*R*[*F* ^2^ > 2σ(*F* ^2^)], *wR*(*F* ^2^), *S*	0.052, 0.153, 1.06	0.063, 0.166, 1.04
No. of reflections	3548	6456
No. of parameters	234	429
No. of restraints	2	4
H-atom treatment	H atoms treated by a mixture of independent and constrained refinement	H atoms treated by a mixture of independent and constrained refinement
Δρ_max_, Δρ_min_ (e Å^−3^)	0.71, −0.27	0.26, −0.27

Computer programs: *SMART* and *SAINT* (Bruker, 2014 ▸), *SHELXT2014* (Sheldrick, 2015*a* ▸), *SHELXL2017* (Sheldrick, 2015*b* ▸) and *X-SEED* (Barbour, 2001 ▸).

## supplementary crystallographic information

### 1,4-Dihydroxy-2-propoxyanthraquinone acetonitrile monosolvate (1) Crystal data

**Table:**

C_17_H_14_O_5_·C_2_H_3_N	*Z* = 2
*M**_r_* = 339.33	*F*(000) = 356
Triclinic, *P*1	*D*_x_ = 1.425 Mg m^−^^3^
*a* = 8.2160 (11) Å	Mo *K*α radiation, λ = 0.71073 Å
*b* = 9.8605 (13) Å	Cell parameters from 3016 reflections
*c* = 10.7410 (14) Å	θ = 2.7–27.2°
α = 95.999 (2)°	µ = 0.10 mm^−^^1^
β = 90.181 (2)°	*T* = 100 K
γ = 113.774 (2)°	Cut irregular block, orange
*V* = 790.99 (18) Å^3^	0.45 × 0.18 × 0.09 mm

### 1,4-Dihydroxy-2-propoxyanthraquinone acetonitrile monosolvate (1) Data collection

**Table:**

Bruker APEXII CCD diffractometer	3548 independent reflections
Radiation source: fine-focus sealed tube	2718 reflections with *I* > 2σ(*I*)
Graphite monochromator	*R*_int_ = 0.022
Detector resolution: 8.3660 pixels mm^-1^	θ_max_ = 27.3°, θ_min_ = 1.9°
phi and ω scans	*h* = −10→10
Absorption correction: multi-scan (SADABS; Bruker, 2014)	*k* = −12→12
*T*_min_ = 0.869, *T*_max_ = 1.000	*l* = −13→13
10216 measured reflections	

### 1,4-Dihydroxy-2-propoxyanthraquinone acetonitrile monosolvate (1) Refinement

**Table:**

Refinement on *F*^2^	2 restraints
Least-squares matrix: full	Hydrogen site location: mixed
*R*[*F*^2^ > 2σ(*F*^2^)] = 0.052	H atoms treated by a mixture of independent and constrained refinement
*wR*(*F*^2^) = 0.153	*w* = 1/[σ^2^(*F*_o_^2^) + (0.0962*P*)^2^ + 0.0684*P*] where *P* = (*F*_o_^2^ + 2*F*_c_^2^)/3
*S* = 1.06	(Δ/σ)_max_ < 0.001
3548 reflections	Δρ_max_ = 0.71 e Å^−^^3^
234 parameters	Δρ_min_ = −0.27 e Å^−^^3^

### 1,4-Dihydroxy-2-propoxyanthraquinone acetonitrile monosolvate (1) Special details

**Table:**

Geometry. All esds (except the esd in the dihedral angle between two l.s. planes) are estimated using the full covariance matrix. The cell esds are taken into account individually in the estimation of esds in distances, angles and torsion angles; correlations between esds in cell parameters are only used when they are defined by crystal symmetry. An approximate (isotropic) treatment of cell esds is used for estimating esds involving l.s. planes.

### 1,4-Dihydroxy-2-propoxyanthraquinone acetonitrile monosolvate (1) Fractional atomic coordinates and isotropic or equivalent isotropic displacement parameters (Å^2^)

**Table:**

	*x*	*y*	*z*	*U*_iso_*/*U*_eq_	
O1	0.93351 (13)	0.54640 (11)	0.31245 (9)	0.0224 (2)	
H1O	0.943 (2)	0.6376 (15)	0.3329 (15)	0.027*	
N1	0.69308 (19)	1.25060 (16)	0.93564 (13)	0.0376 (4)	
C1	0.83016 (17)	0.46925 (15)	0.39909 (12)	0.0179 (3)	
O2	0.85708 (12)	0.26110 (10)	0.29025 (9)	0.0216 (2)	
C2	0.78580 (17)	0.31187 (15)	0.38774 (12)	0.0186 (3)	
O3	0.50110 (13)	0.19718 (10)	0.64607 (9)	0.0231 (3)	
H3O	0.471 (2)	0.2555 (17)	0.6993 (14)	0.028*	
C3	0.67840 (17)	0.22550 (15)	0.47188 (12)	0.0190 (3)	
H3	0.650425	0.121543	0.464073	0.023*	
O4	0.48044 (13)	0.42907 (11)	0.76272 (9)	0.0236 (2)	
C4	0.60915 (17)	0.28978 (15)	0.56995 (12)	0.0184 (3)	
O5	0.90743 (13)	0.77360 (10)	0.43289 (9)	0.0237 (3)	
C5	0.65148 (17)	0.44303 (14)	0.58446 (12)	0.0168 (3)	
C6	0.58069 (17)	0.50732 (15)	0.68671 (12)	0.0186 (3)	
C7	0.63155 (17)	0.67078 (15)	0.70039 (12)	0.0182 (3)	
C8	0.57026 (18)	0.73660 (16)	0.80017 (12)	0.0215 (3)	
H8	0.496307	0.676508	0.858499	0.026*	
C9	0.61668 (18)	0.88946 (16)	0.81480 (13)	0.0230 (3)	
H9	0.577257	0.934122	0.884204	0.028*	
C10	0.72114 (18)	0.97713 (15)	0.72752 (13)	0.0232 (3)	
H10	0.750414	1.081320	0.736488	0.028*	
C11	0.78274 (18)	0.91324 (15)	0.62752 (13)	0.0211 (3)	
H11	0.854261	0.973685	0.568420	0.025*	
C12	0.73939 (17)	0.75953 (14)	0.61376 (12)	0.0175 (3)	
C13	0.80994 (17)	0.69324 (15)	0.50858 (12)	0.0185 (3)	
C14	0.76399 (17)	0.53320 (14)	0.49710 (12)	0.0171 (3)	
C15	0.80376 (18)	0.10079 (14)	0.26995 (12)	0.0197 (3)	
H15A	0.672284	0.048972	0.262662	0.024*	
H15B	0.847744	0.066928	0.341400	0.024*	
C16	0.88231 (18)	0.06548 (15)	0.15066 (12)	0.0216 (3)	
H16A	1.013724	0.117694	0.158502	0.026*	
H16B	0.839009	0.100713	0.079785	0.026*	
C17	0.8285 (2)	−0.10258 (15)	0.12525 (13)	0.0259 (3)	
H17A	0.864454	−0.138135	0.198037	0.039*	
H17B	0.887518	−0.124293	0.051405	0.039*	
H17C	0.699127	−0.153202	0.110002	0.039*	
C18	0.7439 (2)	1.37684 (18)	0.95263 (14)	0.0300 (3)	
C19	0.8085 (2)	1.53842 (19)	0.97699 (19)	0.0451 (5)	
H19A	0.707605	1.567300	0.974361	0.068*	
H19B	0.891236	1.584642	0.913041	0.068*	
H19C	0.870055	1.572114	1.059963	0.068*	

### 1,4-Dihydroxy-2-propoxyanthraquinone acetonitrile monosolvate (1) Atomic displacement parameters (Å^2^)

**Table:**

	*U*^11^	*U*^22^	*U*^33^	*U*^12^	*U*^13^	*U*^23^
O1	0.0257 (5)	0.0171 (5)	0.0230 (5)	0.0076 (4)	0.0075 (4)	0.0008 (4)
N1	0.0451 (8)	0.0342 (8)	0.0356 (8)	0.0199 (7)	0.0016 (6)	−0.0023 (6)
C1	0.0159 (6)	0.0174 (6)	0.0186 (6)	0.0052 (5)	0.0005 (5)	0.0013 (5)
O2	0.0243 (5)	0.0157 (5)	0.0244 (5)	0.0086 (4)	0.0078 (4)	−0.0012 (4)
C2	0.0173 (6)	0.0200 (7)	0.0190 (6)	0.0092 (5)	0.0005 (5)	−0.0026 (5)
O3	0.0278 (5)	0.0164 (5)	0.0249 (5)	0.0086 (4)	0.0091 (4)	0.0029 (4)
C3	0.0188 (6)	0.0151 (6)	0.0230 (7)	0.0071 (5)	0.0002 (5)	0.0003 (5)
O4	0.0263 (5)	0.0202 (5)	0.0239 (5)	0.0092 (4)	0.0069 (4)	0.0018 (4)
C4	0.0176 (6)	0.0184 (6)	0.0194 (6)	0.0076 (5)	0.0011 (5)	0.0021 (5)
O5	0.0271 (5)	0.0170 (5)	0.0247 (5)	0.0066 (4)	0.0067 (4)	0.0018 (4)
C5	0.0165 (6)	0.0167 (6)	0.0170 (6)	0.0071 (5)	−0.0007 (5)	0.0000 (5)
C6	0.0178 (6)	0.0193 (7)	0.0193 (6)	0.0086 (5)	0.0004 (5)	0.0007 (5)
C7	0.0168 (6)	0.0182 (7)	0.0195 (6)	0.0078 (5)	−0.0019 (5)	−0.0012 (5)
C8	0.0225 (7)	0.0233 (7)	0.0192 (6)	0.0106 (6)	0.0007 (5)	−0.0004 (5)
C9	0.0228 (7)	0.0245 (7)	0.0230 (7)	0.0131 (6)	−0.0025 (5)	−0.0056 (5)
C10	0.0235 (7)	0.0183 (7)	0.0282 (7)	0.0107 (6)	−0.0037 (6)	−0.0042 (5)
C11	0.0207 (7)	0.0189 (6)	0.0236 (7)	0.0084 (5)	−0.0014 (5)	0.0008 (5)
C12	0.0167 (6)	0.0173 (7)	0.0180 (6)	0.0073 (5)	−0.0029 (5)	−0.0018 (5)
C13	0.0175 (6)	0.0170 (7)	0.0201 (6)	0.0065 (5)	−0.0009 (5)	0.0006 (5)
C14	0.0161 (6)	0.0156 (7)	0.0185 (6)	0.0061 (5)	−0.0015 (5)	−0.0012 (5)
C15	0.0206 (7)	0.0139 (6)	0.0233 (7)	0.0065 (5)	0.0030 (5)	−0.0017 (5)
C16	0.0223 (7)	0.0212 (7)	0.0208 (7)	0.0089 (5)	0.0021 (5)	−0.0006 (5)
C17	0.0319 (8)	0.0222 (7)	0.0228 (7)	0.0113 (6)	0.0068 (6)	−0.0028 (5)
C18	0.0311 (8)	0.0349 (9)	0.0271 (7)	0.0178 (7)	−0.0004 (6)	−0.0015 (6)
C19	0.0445 (10)	0.0300 (9)	0.0596 (11)	0.0163 (8)	−0.0157 (9)	−0.0045 (8)

### 1,4-Dihydroxy-2-propoxyanthraquinone acetonitrile monosolvate (1) Geometric parameters (Å, º)

**Table:**

O1—C1	1.3410 (15)	C9—C10	1.392 (2)
O1—H1O	0.875 (13)	C9—H9	0.9500
N1—C18	1.136 (2)	C10—C11	1.3877 (19)
C1—C14	1.3938 (19)	C10—H10	0.9500
C1—C2	1.4353 (19)	C11—C12	1.4013 (18)
O2—C2	1.3493 (16)	C11—H11	0.9500
O2—C15	1.4516 (15)	C12—C13	1.4814 (19)
C2—C3	1.3714 (19)	C13—C14	1.4584 (19)
O3—C4	1.3407 (15)	C15—C16	1.5075 (18)
O3—H3O	0.874 (14)	C15—H15A	0.9900
C3—C4	1.4106 (18)	C15—H15B	0.9900
C3—H3	0.9500	C16—C17	1.5265 (18)
O4—C6	1.2505 (16)	C16—H16A	0.9900
C4—C5	1.3983 (18)	C16—H16B	0.9900
O5—C13	1.2480 (16)	C17—H17A	0.9800
C5—C14	1.4292 (19)	C17—H17B	0.9800
C5—C6	1.4498 (18)	C17—H17C	0.9800
C6—C7	1.4836 (18)	C18—C19	1.456 (2)
C7—C8	1.3947 (18)	C19—H19A	0.9800
C7—C12	1.4025 (18)	C19—H19B	0.9800
C8—C9	1.3887 (19)	C19—H19C	0.9800
C8—H8	0.9500		
			
C1—O1—H1O	104.0 (11)	C12—C11—H11	120.0
O1—C1—C14	123.72 (12)	C11—C12—C7	119.64 (12)
O1—C1—C2	117.06 (11)	C11—C12—C13	119.48 (12)
C14—C1—C2	119.22 (12)	C7—C12—C13	120.88 (12)
C2—O2—C15	116.12 (10)	O5—C13—C14	121.35 (12)
O2—C2—C3	125.11 (12)	O5—C13—C12	120.31 (12)
O2—C2—C1	114.62 (12)	C14—C13—C12	118.33 (12)
C3—C2—C1	120.27 (12)	C1—C14—C5	120.48 (12)
C4—O3—H3O	104.0 (11)	C1—C14—C13	119.04 (12)
C2—C3—C4	120.53 (12)	C5—C14—C13	120.47 (12)
C2—C3—H3	119.7	O2—C15—C16	107.99 (10)
C4—C3—H3	119.7	O2—C15—H15A	110.1
O3—C4—C5	122.48 (11)	C16—C15—H15A	110.1
O3—C4—C3	116.90 (12)	O2—C15—H15B	110.1
C5—C4—C3	120.61 (12)	C16—C15—H15B	110.1
C4—C5—C14	118.88 (12)	H15A—C15—H15B	108.4
C4—C5—C6	119.80 (12)	C15—C16—C17	109.75 (11)
C14—C5—C6	121.32 (12)	C15—C16—H16A	109.7
O4—C6—C5	121.78 (12)	C17—C16—H16A	109.7
O4—C6—C7	120.05 (11)	C15—C16—H16B	109.7
C5—C6—C7	118.17 (12)	C17—C16—H16B	109.7
C8—C7—C12	119.70 (12)	H16A—C16—H16B	108.2
C8—C7—C6	119.52 (12)	C16—C17—H17A	109.5
C12—C7—C6	120.79 (12)	C16—C17—H17B	109.5
C9—C8—C7	120.41 (13)	H17A—C17—H17B	109.5
C9—C8—H8	119.8	C16—C17—H17C	109.5
C7—C8—H8	119.8	H17A—C17—H17C	109.5
C8—C9—C10	119.86 (12)	H17B—C17—H17C	109.5
C8—C9—H9	120.1	N1—C18—C19	178.89 (17)
C10—C9—H9	120.1	C18—C19—H19A	109.5
C11—C10—C9	120.40 (12)	C18—C19—H19B	109.5
C11—C10—H10	119.8	H19A—C19—H19B	109.5
C9—C10—H10	119.8	C18—C19—H19C	109.5
C10—C11—C12	119.97 (13)	H19A—C19—H19C	109.5
C10—C11—H11	120.0	H19B—C19—H19C	109.5
			
C15—O2—C2—C3	4.50 (19)	C9—C10—C11—C12	0.2 (2)
C15—O2—C2—C1	−175.30 (10)	C10—C11—C12—C7	0.9 (2)
O1—C1—C2—O2	0.80 (18)	C10—C11—C12—C13	−178.36 (11)
C14—C1—C2—O2	−179.69 (11)	C8—C7—C12—C11	−0.8 (2)
O1—C1—C2—C3	−179.01 (11)	C6—C7—C12—C11	178.81 (11)
C14—C1—C2—C3	0.5 (2)	C8—C7—C12—C13	178.54 (11)
O2—C2—C3—C4	−179.31 (12)	C6—C7—C12—C13	−1.9 (2)
C1—C2—C3—C4	0.5 (2)	C11—C12—C13—O5	0.1 (2)
C2—C3—C4—O3	178.11 (11)	C7—C12—C13—O5	−179.19 (11)
C2—C3—C4—C5	−1.2 (2)	C11—C12—C13—C14	179.48 (11)
O3—C4—C5—C14	−178.34 (11)	C7—C12—C13—C14	0.19 (19)
C3—C4—C5—C14	0.9 (2)	O1—C1—C14—C5	178.71 (11)
O3—C4—C5—C6	1.4 (2)	C2—C1—C14—C5	−0.8 (2)
C3—C4—C5—C6	−179.28 (11)	O1—C1—C14—C13	−0.8 (2)
C4—C5—C6—O4	−0.6 (2)	C2—C1—C14—C13	179.77 (11)
C14—C5—C6—O4	179.13 (11)	C4—C5—C14—C1	0.1 (2)
C4—C5—C6—C7	178.82 (11)	C6—C5—C14—C1	−179.73 (11)
C14—C5—C6—C7	−1.4 (2)	C4—C5—C14—C13	179.51 (11)
O4—C6—C7—C8	1.5 (2)	C6—C5—C14—C13	−0.3 (2)
C5—C6—C7—C8	−177.94 (11)	O5—C13—C14—C1	−0.3 (2)
O4—C6—C7—C12	−178.03 (11)	C12—C13—C14—C1	−179.62 (11)
C5—C6—C7—C12	2.49 (19)	O5—C13—C14—C5	−179.72 (11)
C12—C7—C8—C9	−0.6 (2)	C12—C13—C14—C5	0.91 (19)
C6—C7—C8—C9	179.85 (11)	C2—O2—C15—C16	174.51 (10)
C7—C8—C9—C10	1.7 (2)	O2—C15—C16—C17	−179.69 (10)
C8—C9—C10—C11	−1.5 (2)		

### 1,4-Dihydroxy-2-propoxyanthraquinone acetonitrile monosolvate (1) Hydrogen-bond geometry (Å, º)

**Table:**

*D*—H···*A*	*D*—H	H···*A*	*D*···*A*	*D*—H···*A*
O1—H1*O*···O5	0.88 (1)	1.75 (1)	2.5537 (13)	152 (2)
O3—H3*O*···O4	0.87 (1)	1.75 (1)	2.5578 (13)	153 (2)
C10—H10···N1	0.95	2.73	3.4009 (19)	128
C15—H15*A*···O3^i^	0.99	2.57	3.2179 (16)	123
C11—H11···O5^ii^	0.95	2.47	3.2446 (17)	138

Symmetry codes: (i) −*x*+1, −*y*, −*z*+1; (ii) −*x*+2, −*y*+2, −*z*+1.

### 2-Butoxy-1,4-dihydroxyanthraquinone (2) Crystal data

**Table:**

C_18_H_16_O_5_	*F*(000) = 1312
*M**_r_* = 312.31	*D*_x_ = 1.428 Mg m^−^^3^
Monoclinic, *P*2_1_/*n*	Mo *K*α radiation, λ = 0.71073 Å
*a* = 4.7730 (9) Å	Cell parameters from 2921 reflections
*b* = 44.272 (8) Å	θ = 2.4–23.8°
*c* = 13.807 (3) Å	µ = 0.10 mm^−^^1^
β = 95.164 (2)°	*T* = 100 K
*V* = 2905.8 (9) Å^3^	Cut irregular block, orange-red
*Z* = 8	0.48 × 0.10 × 0.03 mm

### 2-Butoxy-1,4-dihydroxyanthraquinone (2) Data collection

**Table:**

Bruker APEXII CCD diffractometer	6456 independent reflections
Radiation source: fine-focus sealed tube	3747 reflections with *I* > 2σ(*I*)
Graphite monochromator	*R*_int_ = 0.104
Detector resolution: 8.3660 pixels mm^-1^	θ_max_ = 27.2°, θ_min_ = 1.6°
phi and ω scans	*h* = −6→6
Absorption correction: multi-scan (SADABS; Bruker, 2014)	*k* = −56→56
*T*_min_ = 0.854, *T*_max_ = 1.000	*l* = −17→17
36901 measured reflections	

### 2-Butoxy-1,4-dihydroxyanthraquinone (2) Refinement

**Table:**

Refinement on *F*^2^	4 restraints
Least-squares matrix: full	Hydrogen site location: mixed
*R*[*F*^2^ > 2σ(*F*^2^)] = 0.063	H atoms treated by a mixture of independent and constrained refinement
*wR*(*F*^2^) = 0.166	*w* = 1/[σ^2^(*F*_o_^2^) + (0.047*P*)^2^ + 2.0816*P*] where *P* = (*F*_o_^2^ + 2*F*_c_^2^)/3
*S* = 1.04	(Δ/σ)_max_ = 0.001
6456 reflections	Δρ_max_ = 0.26 e Å^−^^3^
429 parameters	Δρ_min_ = −0.27 e Å^−^^3^

### 2-Butoxy-1,4-dihydroxyanthraquinone (2) Special details

**Table:**

Geometry. All esds (except the esd in the dihedral angle between two l.s. planes) are estimated using the full covariance matrix. The cell esds are taken into account individually in the estimation of esds in distances, angles and torsion angles; correlations between esds in cell parameters are only used when they are defined by crystal symmetry. An approximate (isotropic) treatment of cell esds is used for estimating esds involving l.s. planes.

### 2-Butoxy-1,4-dihydroxyanthraquinone (2) Fractional atomic coordinates and isotropic or equivalent isotropic displacement parameters (Å^2^)

**Table:**

	*x*	*y*	*z*	*U*_iso_*/*U*_eq_	
O1	−0.3186 (4)	0.27290 (4)	0.38526 (14)	0.0341 (5)	
H1O	−0.448 (5)	0.2616 (6)	0.359 (2)	0.041*	
C1	−0.3234 (6)	0.29688 (6)	0.32535 (19)	0.0272 (6)	
O2	0.0428 (4)	0.31529 (4)	0.43337 (13)	0.0312 (5)	
C2	−0.1245 (6)	0.32043 (6)	0.3504 (2)	0.0275 (6)	
O3	−0.2823 (4)	0.37359 (4)	0.15477 (15)	0.0350 (5)	
H3O	−0.399 (6)	0.3708 (7)	0.1034 (17)	0.042*	
C3	−0.1160 (6)	0.34561 (6)	0.2928 (2)	0.0287 (6)	
H3	0.017152	0.361103	0.309821	0.034*	
O4	−0.6827 (4)	0.35220 (4)	0.04208 (14)	0.0367 (5)	
C4	−0.3032 (6)	0.34858 (6)	0.2089 (2)	0.0286 (6)	
O5	−0.7188 (4)	0.25237 (4)	0.26716 (15)	0.0375 (5)	
C5	−0.5023 (5)	0.32611 (6)	0.18344 (19)	0.0254 (6)	
O6	0.7609 (4)	0.39771 (4)	0.48700 (15)	0.0355 (5)	
H6O	0.636 (6)	0.3937 (7)	0.4395 (17)	0.043*	
C6	−0.6920 (6)	0.32954 (6)	0.0958 (2)	0.0293 (6)	
O7	1.1866 (4)	0.42067 (4)	0.59154 (14)	0.0367 (5)	
C7	−0.9003 (5)	0.30521 (6)	0.07061 (19)	0.0266 (6)	
O8	1.2269 (4)	0.50371 (4)	0.37289 (15)	0.0341 (5)	
H8O	1.123 (6)	0.5094 (7)	0.3208 (16)	0.041*	
C8	−1.0870 (6)	0.30801 (6)	−0.0124 (2)	0.0328 (7)	
H8	−1.081236	0.325412	−0.052455	0.039*	
O9	0.8632 (4)	0.50517 (4)	0.22553 (14)	0.0359 (5)	
C9	−1.2818 (6)	0.28529 (7)	−0.0366 (2)	0.0349 (7)	
H9	−1.410599	0.287331	−0.092840	0.042*	
O10	0.4078 (4)	0.40025 (4)	0.33801 (14)	0.0346 (5)	
C10	−1.2893 (6)	0.25958 (6)	0.0210 (2)	0.0340 (7)	
H10	−1.419875	0.243897	0.003368	0.041*	
C11	−1.1065 (6)	0.25689 (6)	0.1040 (2)	0.0321 (7)	
H11	−1.114821	0.239564	0.144244	0.038*	
C12	−0.9090 (6)	0.27958 (6)	0.12909 (19)	0.0274 (6)	
C13	−0.7114 (6)	0.27581 (6)	0.2171 (2)	0.0286 (6)	
C14	−0.5100 (5)	0.29996 (6)	0.2427 (2)	0.0264 (6)	
C15	0.2386 (6)	0.33909 (6)	0.4653 (2)	0.0313 (6)	
H15B	0.136313	0.358244	0.473426	0.038*	
H15A	0.375876	0.342303	0.416602	0.038*	
C16	0.3885 (6)	0.32930 (6)	0.5611 (2)	0.0339 (7)	
H16A	0.538850	0.344065	0.580570	0.041*	
H16B	0.479215	0.309504	0.552161	0.041*	
C17	0.1962 (7)	0.32659 (7)	0.6425 (2)	0.0402 (7)	
H17A	0.060155	0.310046	0.626768	0.048*	
H17B	0.088094	0.345572	0.646233	0.048*	
C18	0.3532 (8)	0.32039 (7)	0.7413 (2)	0.0488 (9)	
H18A	0.475930	0.302797	0.736548	0.073*	
H18B	0.217716	0.316330	0.788912	0.073*	
H18C	0.467288	0.338036	0.761934	0.073*	
C19	0.8649 (6)	0.42371 (6)	0.4540 (2)	0.0296 (6)	
C20	1.0975 (6)	0.43683 (6)	0.5119 (2)	0.0301 (6)	
C21	1.2100 (6)	0.46359 (6)	0.4840 (2)	0.0302 (6)	
H21	1.362242	0.472501	0.522992	0.036*	
C22	1.1009 (6)	0.47784 (6)	0.3981 (2)	0.0300 (6)	
C23	0.8739 (6)	0.46552 (6)	0.3399 (2)	0.0278 (6)	
C24	0.7634 (6)	0.48070 (6)	0.2524 (2)	0.0293 (6)	
C25	0.5272 (6)	0.46627 (6)	0.1918 (2)	0.0300 (6)	
C26	0.4175 (6)	0.48005 (7)	0.1061 (2)	0.0365 (7)	
H26	0.489850	0.498971	0.087551	0.044*	
C27	0.2030 (7)	0.46627 (7)	0.0475 (2)	0.0413 (8)	
H27	0.132225	0.475545	−0.011761	0.050*	
C28	0.0917 (6)	0.43906 (7)	0.0752 (2)	0.0394 (7)	
H28	−0.057104	0.429841	0.035422	0.047*	
C29	0.1970 (6)	0.42527 (6)	0.1608 (2)	0.0354 (7)	
H29	0.120415	0.406580	0.179632	0.042*	
C30	0.4146 (6)	0.43869 (6)	0.2195 (2)	0.0305 (6)	
C31	0.5203 (6)	0.42406 (6)	0.3111 (2)	0.0296 (6)	
C32	0.7571 (6)	0.43774 (6)	0.3698 (2)	0.0290 (6)	
C33	1.4201 (6)	0.43232 (6)	0.6555 (2)	0.0354 (7)	
H33A	1.359002	0.449853	0.692861	0.042*	
H33B	1.575349	0.438816	0.617335	0.042*	
C34	1.5157 (7)	0.40682 (7)	0.7235 (2)	0.0392 (7)	
H34A	1.589349	0.390176	0.685154	0.047*	
H34B	1.351732	0.398995	0.754780	0.047*	
C35	1.7415 (7)	0.41641 (7)	0.8018 (2)	0.0449 (8)	
H35A	1.663659	0.431719	0.844235	0.054*	
H35B	1.900044	0.425732	0.771114	0.054*	
C36	1.8488 (8)	0.38931 (8)	0.8632 (2)	0.0525 (9)	
H36A	1.695632	0.381183	0.898166	0.079*	
H36B	2.003713	0.395739	0.910094	0.079*	
H36C	1.915812	0.373660	0.820721	0.079*	

### 2-Butoxy-1,4-dihydroxyanthraquinone (2) Atomic displacement parameters (Å^2^)

**Table:**

	*U*^11^	*U*^22^	*U*^33^	*U*^12^	*U*^13^	*U*^23^
O1	0.0317 (11)	0.0275 (11)	0.0412 (12)	−0.0051 (9)	−0.0072 (9)	0.0049 (9)
C1	0.0237 (14)	0.0243 (14)	0.0334 (15)	0.0016 (11)	0.0025 (12)	0.0025 (12)
O2	0.0262 (10)	0.0271 (10)	0.0389 (11)	−0.0023 (8)	−0.0038 (9)	−0.0001 (8)
C2	0.0236 (14)	0.0239 (14)	0.0350 (15)	0.0027 (11)	0.0020 (12)	−0.0028 (11)
O3	0.0343 (12)	0.0276 (11)	0.0423 (12)	−0.0038 (9)	−0.0007 (9)	0.0058 (9)
C3	0.0229 (14)	0.0232 (14)	0.0400 (16)	−0.0018 (11)	0.0033 (12)	−0.0031 (12)
O4	0.0353 (12)	0.0298 (11)	0.0446 (12)	0.0010 (9)	0.0009 (9)	0.0078 (9)
C4	0.0251 (15)	0.0226 (14)	0.0387 (16)	0.0024 (11)	0.0057 (12)	0.0020 (12)
O5	0.0347 (12)	0.0297 (11)	0.0464 (12)	−0.0070 (9)	−0.0060 (9)	0.0081 (9)
C5	0.0211 (14)	0.0233 (13)	0.0325 (15)	0.0019 (11)	0.0059 (11)	−0.0008 (11)
O6	0.0331 (12)	0.0261 (10)	0.0468 (13)	−0.0057 (9)	0.0015 (9)	0.0027 (9)
C6	0.0236 (14)	0.0279 (15)	0.0364 (16)	0.0066 (11)	0.0024 (12)	0.0021 (12)
O7	0.0321 (11)	0.0302 (11)	0.0467 (12)	−0.0039 (9)	−0.0022 (9)	0.0032 (9)
C7	0.0224 (14)	0.0272 (14)	0.0306 (15)	0.0048 (11)	0.0048 (12)	−0.0013 (11)
O8	0.0304 (11)	0.0261 (10)	0.0456 (12)	−0.0053 (9)	0.0028 (9)	0.0013 (9)
C8	0.0322 (16)	0.0337 (16)	0.0324 (16)	0.0068 (13)	0.0024 (13)	0.0022 (12)
O9	0.0342 (12)	0.0263 (11)	0.0474 (12)	−0.0037 (9)	0.0047 (9)	0.0027 (9)
C9	0.0273 (16)	0.0401 (17)	0.0360 (17)	0.0071 (13)	−0.0035 (13)	−0.0072 (13)
O10	0.0291 (11)	0.0263 (11)	0.0487 (12)	−0.0046 (8)	0.0049 (9)	−0.0003 (9)
C10	0.0279 (16)	0.0323 (16)	0.0411 (17)	−0.0001 (12)	−0.0006 (13)	−0.0099 (13)
C11	0.0292 (16)	0.0276 (15)	0.0394 (16)	−0.0003 (12)	0.0030 (13)	−0.0039 (12)
C12	0.0235 (14)	0.0266 (14)	0.0323 (15)	0.0036 (11)	0.0029 (12)	−0.0039 (12)
C13	0.0239 (14)	0.0282 (15)	0.0335 (15)	0.0013 (12)	0.0008 (12)	−0.0015 (12)
C14	0.0207 (13)	0.0232 (14)	0.0355 (15)	0.0006 (11)	0.0033 (11)	−0.0004 (11)
C15	0.0266 (15)	0.0225 (14)	0.0443 (17)	−0.0034 (11)	0.0009 (13)	−0.0019 (12)
C16	0.0295 (16)	0.0277 (15)	0.0427 (17)	−0.0001 (12)	−0.0065 (13)	−0.0047 (13)
C17	0.0374 (18)	0.0405 (18)	0.0415 (18)	0.0057 (14)	−0.0024 (14)	−0.0005 (14)
C18	0.061 (2)	0.0373 (19)	0.0463 (19)	0.0090 (16)	−0.0058 (17)	−0.0034 (15)
C19	0.0277 (15)	0.0196 (13)	0.0425 (17)	−0.0021 (11)	0.0083 (13)	−0.0014 (12)
C20	0.0263 (15)	0.0263 (14)	0.0382 (16)	0.0031 (12)	0.0058 (13)	−0.0011 (12)
C21	0.0243 (15)	0.0255 (14)	0.0412 (17)	−0.0009 (11)	0.0058 (13)	−0.0041 (12)
C22	0.0265 (15)	0.0216 (14)	0.0432 (17)	−0.0018 (11)	0.0107 (13)	−0.0056 (12)
C23	0.0235 (14)	0.0217 (14)	0.0386 (16)	0.0013 (11)	0.0058 (12)	−0.0025 (12)
C24	0.0236 (14)	0.0238 (14)	0.0413 (16)	0.0001 (11)	0.0069 (12)	−0.0037 (12)
C25	0.0247 (15)	0.0257 (14)	0.0404 (16)	0.0012 (11)	0.0064 (12)	−0.0027 (12)
C26	0.0330 (17)	0.0319 (16)	0.0445 (18)	−0.0002 (13)	0.0029 (14)	0.0034 (13)
C27	0.0371 (18)	0.0395 (18)	0.0462 (19)	0.0042 (14)	−0.0024 (15)	0.0013 (14)
C28	0.0311 (17)	0.0384 (17)	0.0475 (19)	0.0008 (13)	−0.0022 (14)	−0.0066 (14)
C29	0.0277 (16)	0.0303 (16)	0.0483 (18)	−0.0006 (12)	0.0049 (14)	−0.0055 (13)
C30	0.0240 (15)	0.0278 (15)	0.0401 (17)	0.0020 (11)	0.0055 (13)	−0.0051 (12)
C31	0.0250 (15)	0.0234 (14)	0.0416 (17)	0.0023 (11)	0.0095 (13)	−0.0047 (12)
C32	0.0227 (14)	0.0242 (14)	0.0405 (16)	0.0004 (11)	0.0059 (12)	−0.0039 (12)
C33	0.0313 (16)	0.0294 (15)	0.0446 (18)	−0.0043 (13)	−0.0009 (13)	−0.0040 (13)
C34	0.0383 (18)	0.0347 (17)	0.0440 (18)	0.0003 (14)	−0.0001 (14)	0.0010 (14)
C35	0.046 (2)	0.0380 (18)	0.0495 (19)	0.0035 (15)	−0.0053 (16)	−0.0038 (15)
C36	0.056 (2)	0.047 (2)	0.052 (2)	0.0065 (17)	−0.0088 (17)	−0.0009 (16)

### 2-Butoxy-1,4-dihydroxyanthraquinone (2) Geometric parameters (Å, º)

**Table:**

O1—C1	1.345 (3)	C16—H16A	0.9900
O1—H1O	0.848 (18)	C16—H16B	0.9900
C1—C14	1.390 (4)	C17—C18	1.520 (4)
C1—C2	1.431 (4)	C17—H17A	0.9900
O2—C2	1.356 (3)	C17—H17B	0.9900
O2—C15	1.450 (3)	C18—H18A	0.9800
C2—C3	1.372 (4)	C18—H18B	0.9800
O3—C4	1.344 (3)	C18—H18C	0.9800
O3—H3O	0.870 (17)	C19—C32	1.377 (4)
C3—C4	1.404 (4)	C19—C20	1.432 (4)
C3—H3	0.9500	C20—C21	1.370 (4)
O4—C6	1.251 (3)	C21—C22	1.402 (4)
C4—C5	1.399 (4)	C21—H21	0.9500
O5—C13	1.249 (3)	C22—C23	1.400 (4)
C5—C14	1.420 (4)	C23—C32	1.426 (4)
C5—C6	1.453 (4)	C23—C24	1.441 (4)
O6—C19	1.349 (3)	C24—C25	1.486 (4)
O6—H6O	0.863 (18)	C25—C26	1.392 (4)
C6—C7	1.485 (4)	C25—C30	1.401 (4)
O7—C20	1.348 (3)	C26—C27	1.388 (4)
O7—C33	1.453 (3)	C26—H26	0.9500
C7—C8	1.393 (4)	C27—C28	1.384 (4)
C7—C12	1.396 (4)	C27—H27	0.9500
O8—C22	1.354 (3)	C28—C29	1.384 (4)
O8—H8O	0.874 (17)	C28—H28	0.9500
C8—C9	1.390 (4)	C29—C30	1.392 (4)
C8—H8	0.9500	C29—H29	0.9500
O9—C24	1.253 (3)	C30—C31	1.469 (4)
C9—C10	1.391 (4)	C31—C32	1.462 (4)
C9—H9	0.9500	C33—C34	1.512 (4)
O10—C31	1.254 (3)	C33—H33A	0.9900
C10—C11	1.381 (4)	C33—H33B	0.9900
C10—H10	0.9500	C34—C35	1.517 (4)
C11—C12	1.399 (4)	C34—H34A	0.9900
C11—H11	0.9500	C34—H34B	0.9900
C12—C13	1.479 (4)	C35—C36	1.531 (4)
C13—C14	1.460 (4)	C35—H35A	0.9900
C15—C16	1.510 (4)	C35—H35B	0.9900
C15—H15B	0.9900	C36—H36A	0.9800
C15—H15A	0.9900	C36—H36B	0.9800
C16—C17	1.518 (4)	C36—H36C	0.9800
			
C1—O1—H1O	103 (2)	C17—C18—H18C	109.5
O1—C1—C14	123.8 (2)	H18A—C18—H18C	109.5
O1—C1—C2	116.9 (2)	H18B—C18—H18C	109.5
C14—C1—C2	119.3 (2)	O6—C19—C32	123.3 (2)
C2—O2—C15	116.7 (2)	O6—C19—C20	116.6 (2)
O2—C2—C3	125.4 (2)	C32—C19—C20	120.1 (2)
O2—C2—C1	114.2 (2)	O7—C20—C21	125.8 (3)
C3—C2—C1	120.4 (2)	O7—C20—C19	114.4 (2)
C4—O3—H3O	105 (2)	C21—C20—C19	119.9 (3)
C2—C3—C4	120.3 (2)	C20—C21—C22	120.1 (3)
C2—C3—H3	119.9	C20—C21—H21	120.0
C4—C3—H3	119.9	C22—C21—H21	120.0
O3—C4—C5	121.9 (2)	O8—C22—C23	121.4 (3)
O3—C4—C3	117.5 (2)	O8—C22—C21	117.3 (2)
C5—C4—C3	120.5 (2)	C23—C22—C21	121.3 (2)
C4—C5—C14	119.2 (2)	C22—C23—C32	118.2 (2)
C4—C5—C6	119.6 (2)	C22—C23—C24	120.4 (2)
C14—C5—C6	121.2 (2)	C32—C23—C24	121.4 (2)
C19—O6—H6O	100 (2)	O9—C24—C23	122.1 (3)
O4—C6—C5	121.7 (2)	O9—C24—C25	119.7 (3)
O4—C6—C7	120.1 (2)	C23—C24—C25	118.2 (2)
C5—C6—C7	118.2 (2)	C26—C25—C30	119.3 (3)
C20—O7—C33	118.4 (2)	C26—C25—C24	119.9 (3)
C8—C7—C12	119.9 (3)	C30—C25—C24	120.8 (3)
C8—C7—C6	119.6 (2)	C27—C26—C25	120.3 (3)
C12—C7—C6	120.6 (2)	C27—C26—H26	119.8
C22—O8—H8O	103 (2)	C25—C26—H26	119.8
C9—C8—C7	119.9 (3)	C28—C27—C26	120.2 (3)
C9—C8—H8	120.1	C28—C27—H27	119.9
C7—C8—H8	120.1	C26—C27—H27	119.9
C8—C9—C10	120.4 (3)	C27—C28—C29	120.1 (3)
C8—C9—H9	119.8	C27—C28—H28	120.0
C10—C9—H9	119.8	C29—C28—H28	120.0
C11—C10—C9	119.9 (3)	C28—C29—C30	120.2 (3)
C11—C10—H10	120.1	C28—C29—H29	119.9
C9—C10—H10	120.1	C30—C29—H29	119.9
C10—C11—C12	120.3 (3)	C29—C30—C25	119.9 (3)
C10—C11—H11	119.9	C29—C30—C31	119.5 (3)
C12—C11—H11	119.9	C25—C30—C31	120.6 (3)
C7—C12—C11	119.7 (2)	O10—C31—C32	121.0 (3)
C7—C12—C13	121.1 (2)	O10—C31—C30	120.2 (2)
C11—C12—C13	119.2 (2)	C32—C31—C30	118.9 (2)
O5—C13—C14	121.7 (2)	C19—C32—C23	120.5 (2)
O5—C13—C12	120.0 (2)	C19—C32—C31	119.5 (2)
C14—C13—C12	118.2 (2)	C23—C32—C31	120.0 (2)
C1—C14—C5	120.3 (2)	O7—C33—C34	106.5 (2)
C1—C14—C13	119.1 (2)	O7—C33—H33A	110.4
C5—C14—C13	120.6 (2)	C34—C33—H33A	110.4
O2—C15—C16	107.4 (2)	O7—C33—H33B	110.4
O2—C15—H15B	110.2	C34—C33—H33B	110.4
C16—C15—H15B	110.2	H33A—C33—H33B	108.6
O2—C15—H15A	110.2	C33—C34—C35	112.9 (2)
C16—C15—H15A	110.2	C33—C34—H34A	109.0
H15B—C15—H15A	108.5	C35—C34—H34A	109.0
C15—C16—C17	113.7 (2)	C33—C34—H34B	109.0
C15—C16—H16A	108.8	C35—C34—H34B	109.0
C17—C16—H16A	108.8	H34A—C34—H34B	107.8
C15—C16—H16B	108.8	C34—C35—C36	110.9 (3)
C17—C16—H16B	108.8	C34—C35—H35A	109.5
H16A—C16—H16B	107.7	C36—C35—H35A	109.5
C16—C17—C18	113.3 (3)	C34—C35—H35B	109.5
C16—C17—H17A	108.9	C36—C35—H35B	109.5
C18—C17—H17A	108.9	H35A—C35—H35B	108.1
C16—C17—H17B	108.9	C35—C36—H36A	109.5
C18—C17—H17B	108.9	C35—C36—H36B	109.5
H17A—C17—H17B	107.7	H36A—C36—H36B	109.5
C17—C18—H18A	109.5	C35—C36—H36C	109.5
C17—C18—H18B	109.5	H36A—C36—H36C	109.5
H18A—C18—H18B	109.5	H36B—C36—H36C	109.5
			
C15—O2—C2—C3	2.6 (4)	C33—O7—C20—C21	0.9 (4)
C15—O2—C2—C1	−176.8 (2)	C33—O7—C20—C19	−179.4 (2)
O1—C1—C2—O2	−0.6 (3)	O6—C19—C20—O7	1.7 (4)
C14—C1—C2—O2	178.7 (2)	C32—C19—C20—O7	−178.9 (2)
O1—C1—C2—C3	179.9 (2)	O6—C19—C20—C21	−178.5 (2)
C14—C1—C2—C3	−0.7 (4)	C32—C19—C20—C21	0.8 (4)
O2—C2—C3—C4	−179.2 (2)	O7—C20—C21—C22	178.6 (3)
C1—C2—C3—C4	0.2 (4)	C19—C20—C21—C22	−1.1 (4)
C2—C3—C4—O3	−179.2 (2)	C20—C21—C22—O8	−177.9 (2)
C2—C3—C4—C5	0.6 (4)	C20—C21—C22—C23	1.0 (4)
O3—C4—C5—C14	178.8 (2)	O8—C22—C23—C32	178.2 (2)
C3—C4—C5—C14	−1.0 (4)	C21—C22—C23—C32	−0.7 (4)
O3—C4—C5—C6	0.4 (4)	O8—C22—C23—C24	−1.7 (4)
C3—C4—C5—C6	−179.4 (2)	C21—C22—C23—C24	179.4 (3)
C4—C5—C6—O4	−0.1 (4)	C22—C23—C24—O9	−0.4 (4)
C14—C5—C6—O4	−178.5 (3)	C32—C23—C24—O9	179.7 (3)
C4—C5—C6—C7	179.8 (2)	C22—C23—C24—C25	178.4 (3)
C14—C5—C6—C7	1.4 (4)	C32—C23—C24—C25	−1.5 (4)
O4—C6—C7—C8	−0.8 (4)	O9—C24—C25—C26	−0.3 (4)
C5—C6—C7—C8	179.3 (2)	C23—C24—C25—C26	−179.1 (3)
O4—C6—C7—C12	178.8 (3)	O9—C24—C25—C30	179.0 (3)
C5—C6—C7—C12	−1.0 (4)	C23—C24—C25—C30	0.2 (4)
C12—C7—C8—C9	0.1 (4)	C30—C25—C26—C27	−1.4 (4)
C6—C7—C8—C9	179.8 (3)	C24—C25—C26—C27	178.0 (3)
C7—C8—C9—C10	−0.8 (4)	C25—C26—C27—C28	1.6 (5)
C8—C9—C10—C11	1.5 (4)	C26—C27—C28—C29	−0.9 (5)
C9—C10—C11—C12	−1.6 (4)	C27—C28—C29—C30	0.1 (4)
C8—C7—C12—C11	−0.2 (4)	C28—C29—C30—C25	0.1 (4)
C6—C7—C12—C11	−179.9 (2)	C28—C29—C30—C31	178.8 (3)
C8—C7—C12—C13	179.3 (2)	C26—C25—C30—C29	0.6 (4)
C6—C7—C12—C13	−0.3 (4)	C24—C25—C30—C29	−178.8 (3)
C10—C11—C12—C7	1.0 (4)	C26—C25—C30—C31	−178.2 (3)
C10—C11—C12—C13	−178.6 (2)	C24—C25—C30—C31	2.5 (4)
C7—C12—C13—O5	−178.4 (3)	C29—C30—C31—O10	−2.8 (4)
C11—C12—C13—O5	1.2 (4)	C25—C30—C31—O10	176.0 (2)
C7—C12—C13—C14	1.3 (4)	C29—C30—C31—C32	177.3 (3)
C11—C12—C13—C14	−179.1 (2)	C25—C30—C31—C32	−3.9 (4)
O1—C1—C14—C5	179.7 (2)	O6—C19—C32—C23	178.8 (2)
C2—C1—C14—C5	0.4 (4)	C20—C19—C32—C23	−0.5 (4)
O1—C1—C14—C13	−1.0 (4)	O6—C19—C32—C31	−0.9 (4)
C2—C1—C14—C13	179.6 (2)	C20—C19—C32—C31	179.8 (2)
C4—C5—C14—C1	0.5 (4)	C22—C23—C32—C19	0.5 (4)
C6—C5—C14—C1	178.9 (2)	C24—C23—C32—C19	−179.6 (3)
C4—C5—C14—C13	−178.8 (2)	C22—C23—C32—C31	−179.9 (2)
C6—C5—C14—C13	−0.4 (4)	C24—C23—C32—C31	0.0 (4)
O5—C13—C14—C1	−0.6 (4)	O10—C31—C32—C19	2.4 (4)
C12—C13—C14—C1	179.7 (2)	C30—C31—C32—C19	−177.7 (2)
O5—C13—C14—C5	178.7 (3)	O10—C31—C32—C23	−177.2 (2)
C12—C13—C14—C5	−1.0 (4)	C30—C31—C32—C23	2.7 (4)
C2—O2—C15—C16	176.7 (2)	C20—O7—C33—C34	−167.3 (2)
O2—C15—C16—C17	−65.1 (3)	O7—C33—C34—C35	−174.3 (3)
C15—C16—C17—C18	−173.1 (2)	C33—C34—C35—C36	−175.2 (3)

### 2-Butoxy-1,4-dihydroxyanthraquinone (2) Hydrogen-bond geometry (Å, º)

**Table:**

*D*—H···*A*	*D*—H	H···*A*	*D*···*A*	*D*—H···*A*
O1—H1*O*···O5	0.85 (2)	1.78 (2)	2.564 (3)	153 (3)
O3—H3*O*···O4	0.87 (2)	1.74 (2)	2.536 (3)	151 (3)
O6—H6*O*···O10	0.86 (2)	1.72 (2)	2.542 (3)	158 (3)
O8—H8*O*···O9	0.87 (2)	1.73 (2)	2.554 (3)	155 (3)
C3—H3···O10	0.95	2.55	3.494 (3)	173
C29—H29···O3	0.95	2.41	3.231 (4)	144
C15—H15*B*···O6^i^	0.99	2.52	3.485 (3)	164
C21—H21···O8^ii^	0.95	2.55	3.502 (3)	180
C33—H33*A*···O9^iii^	0.99	2.56	3.547 (4)	172

Symmetry codes: (i) *x*−1, *y*, *z*; (ii) −*x*+3, −*y*+1, −*z*+1; (iii) −*x*+2, −*y*+1, −*z*+1.
